# Different Dental Students’ Attitude toward General Surgery Subject?

**DOI:** 10.12688/f1000research.163454.1

**Published:** 2025-05-27

**Authors:** Ali Jabbar Alsheakh, Najih Alaridhy, Saif Anmar Badran, Faaiz Alhamdani

**Affiliations:** 1 Dentistry Department, Al Yarmouk University College, Diyala, Iraq; 2 Dental Technologies, Alshaab University, Baghdad, Iraq; 3 Ibn Sina University for Medical and Pharmaceutical Sciences, Baghdad, Baghdad Governorate, Iraq

**Keywords:** Dental health, biomedical knowledge, General surgery

## Abstract

**Background:**

Dental schools may adopt different educational approaches in relation to general surgery subjects. In Iraqi Dental Schools, general surgery subjects are usually given to fourth-year students. To the best of our knowledge, no previous study has investigated undergraduate dental students’ attitudes toward different aspects of this subject.

**Aims:**

To determine the influence of general surgery on oral surgery subjects and students’ preference for the lecturer’s specialty.

**Materials and methods:**

A Google questionnaire of the three main domains was circulated to fourth- and fifth-year dental students in three dental schools. These students were given lectures on general surgery by either a physician or oral surgeon.

**Results:**

Two hundred sixty-five students participated in this study; the majority of responses were positive for general surgery subjects. The chi-square Test showed a highly significant relationship (P=0.000) between the relevance of GS to OS and the extent to which general surgery help in understanding oral surgery. The same test showed a significant relationship (P=0.021) between willingness to become an OMF surgeon and the wish to be given a GS subject by an oral surgeon.

**Conclusion:**

General surgery enjoys a reasonable level of popularity among undergraduate senior dental students. This popularity seems to have influenced undergraduate students’ willingness to become oral and maxillofacial surgeons. This, in turn, made students prefer general surgery subjects given by an oral surgeon.

## Introduction

Dental health is inseparable from general medical health. Dental schools incorporate relevant biomedical knowledge to prepare proficient dentists with efficient medical knowledge. ^1^ This aims to integrate the three pillars of dental education: technical skills, clinical knowledge, and motivation, empathy with patients, critical reasoning, clinical reasoning, and decision-making. ^2– 3^ Accordingly, the dental curriculum includes almost the same basic biological and medical knowledge adopted in medical schools, with more focus on the head and neck region and dental apparatus in particular. Dentistry is unique among medical profession. ^4– 5^ Of the given subjects for undergraduate dental students are general medicine, and general surgery. General medicine is related to a variety of systemic diseases that have a direct or indirect influence on patients undergoing dental treatment. General surgery, on the other hand, attempts to provide dental students with important surgical knowledge that is relevant to their medical background and oral surgical practice. ^6– 7^ Different dental schools may adopt different educational approaches in relation to general surgical subjects. In Iraqi dental schools, general surgery subjects are usually given to fourth-year year students. ^8^ To the best of our knowledge, no previous study has investigated undergraduate dental students’ attitudes toward different aspects of this subject.

### Aim of the study

To determine undergraduate dental students’ preference for general surgery, the study objectives were as follows:

• To determine the influence of general surgery subject on oral surgery subject.
• Student’s preference toward the specialty of the lecturer.

## Materials and methods

This cross-sectional study was approved by the Ethics Committee of Ibn Sina University of Medical and Pharmaceutical Sciences (ISU.16.1.24). This is a questionnaire-based study using an online Google form. This questionnaire was circulated to fourth- and fifth-year dental students at three dental schools. The questionnaire was developed by the first author and revised by the second author to ensure relevance of the questions to the participants. The choice of participants was based on the academic staff who gave GS for the fourth year. Fourth- and fifth-year students were included, as fifth-year students might have had additional input. Fifth-year students are given theoretical lectures mainly on maxillofacial surgery items, which expand their knowledge and appreciation of GS subjects. The questionnaire items covered different aspects of general surgery GS subjects in relation to OS knowledge and practice. The questionnaire items were divided into three domains: the demographic domain, which includes general information about the participants. The second domain is related to the GS subject, whereas the third domain focuses on the relationship between the GS and OS.

## Results

Two hundred sixty-five students participated in this questionnaire-based study. Two-thirds of respondents were females. Males constituted the remaining one-third. Students from three Iraqi dental schools participated in this study. The two dental schools are private dental schools (Al-Yarmouk University college, and Al-Hikmah University College), and one governmental dental school (Ibn Sina University of Medical and Pharmaceutical Sciences. All three dental schools based in Baghdad, Iraq, and Table 1 provide the demographics of the study.

**Table 1.** Study demographic data.

	No.	%
Students’ gender		
Male	84	32.8
Female	172	67.2
Students’ academic year		
Fourth year	160	62.5
Fifth year	96	37.5
Dental school		
Ibn Sina University	95	37.1
Al-Yarmouk UC	76	29.7
Al-Hikmah UC	85	33.2
Academic staff specialty		
Oral surgeon	164	64.1
Physician	92	35.9
Who should give GS lecture		
Oral surgeon	175	68.4
Physician	81	31.6
The wish to be OMFS		
YES	177	69.1
NO	79	30.9

**Figure 1.**
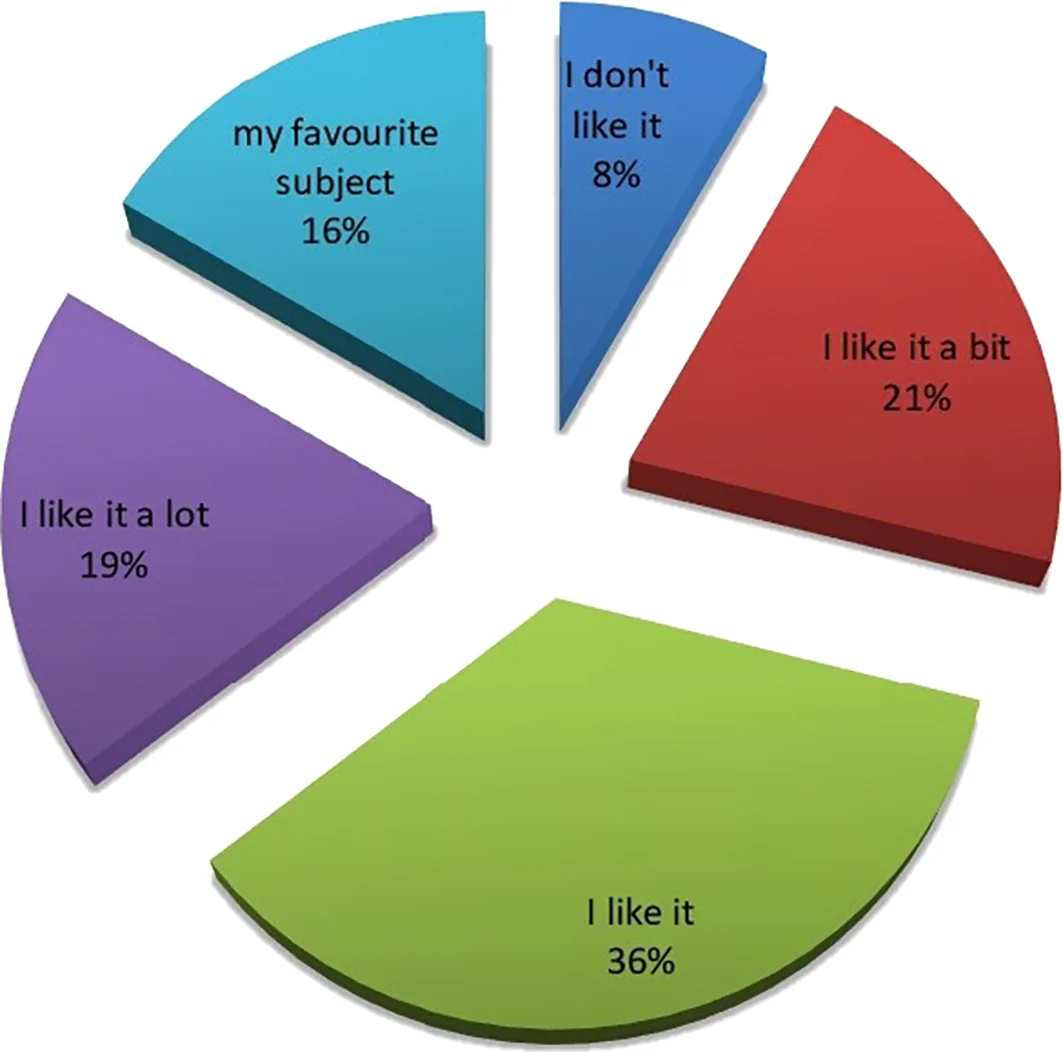
Level of likeness of general surgery.

As shown in Figure 1, students’ preference for GS varies. However, the majority of the responses were positive. There are even students who consider the GS subject to be their favorite. A small minority of students did not seem to like the subject. A comparison between the percentages of responses “my favorite subject” and “I don’t like it” might hint at the likeliness of the subject.

**Figure 2.**
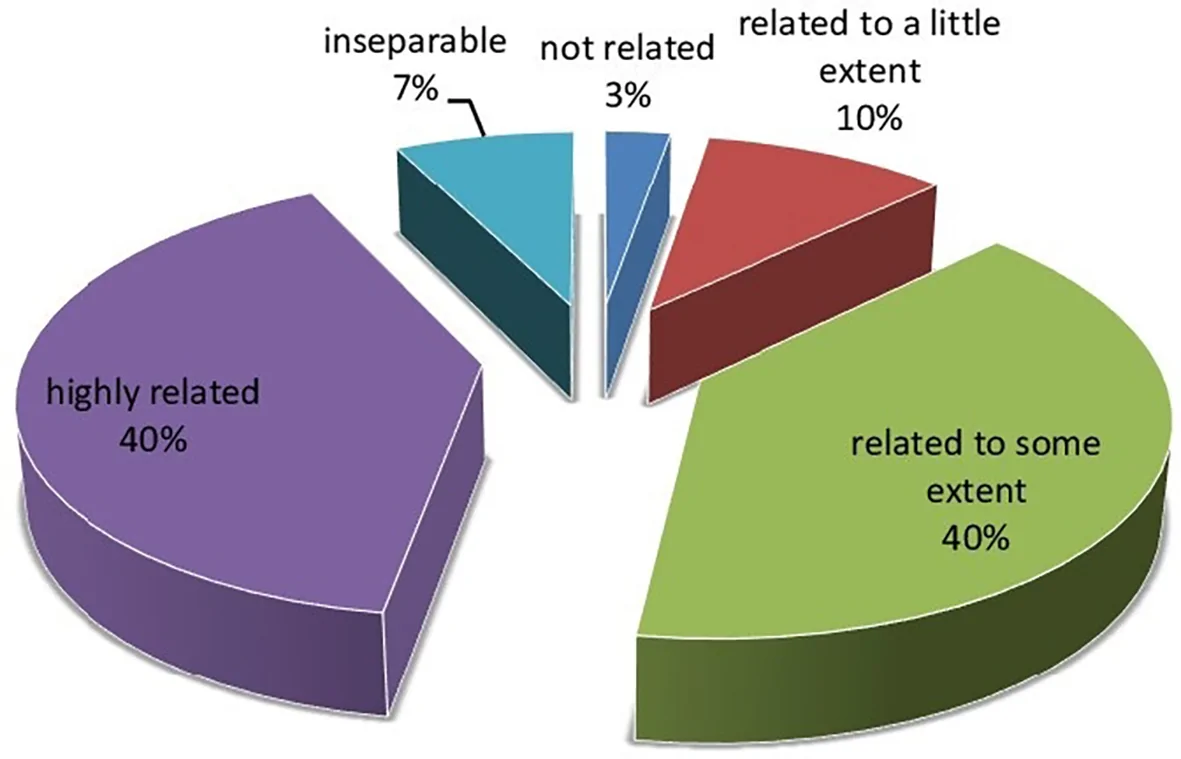
General surgery subject relevance to oral surgery subject.

An overwhelming majority of the students thought GS was related to oral surgery, albeit to a variable degree. Figure 2 also shows that almost half of the students believed in the high relationship between the subjects, and a very small minority of students found GS irrelevant to OS.

**Figure 3.**
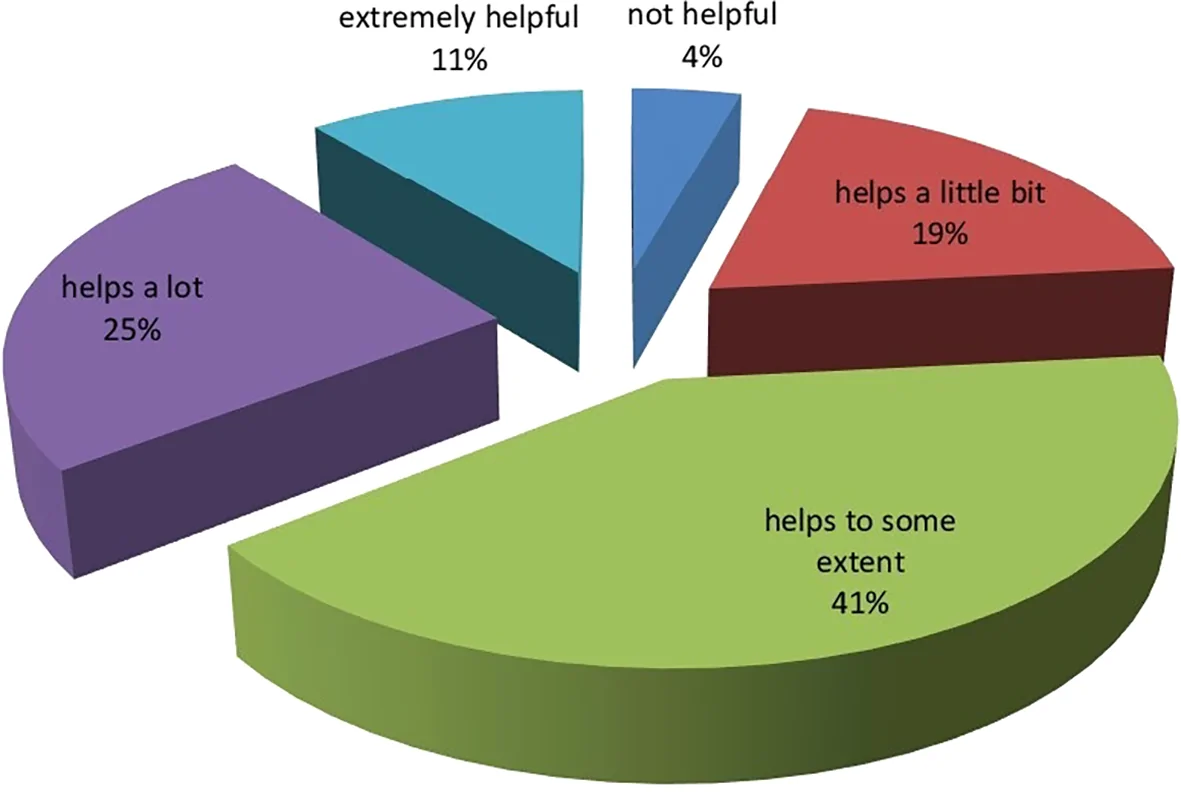
Perceived help provided by general surgery subject in understanding oral surgery.

**Figure 4.**
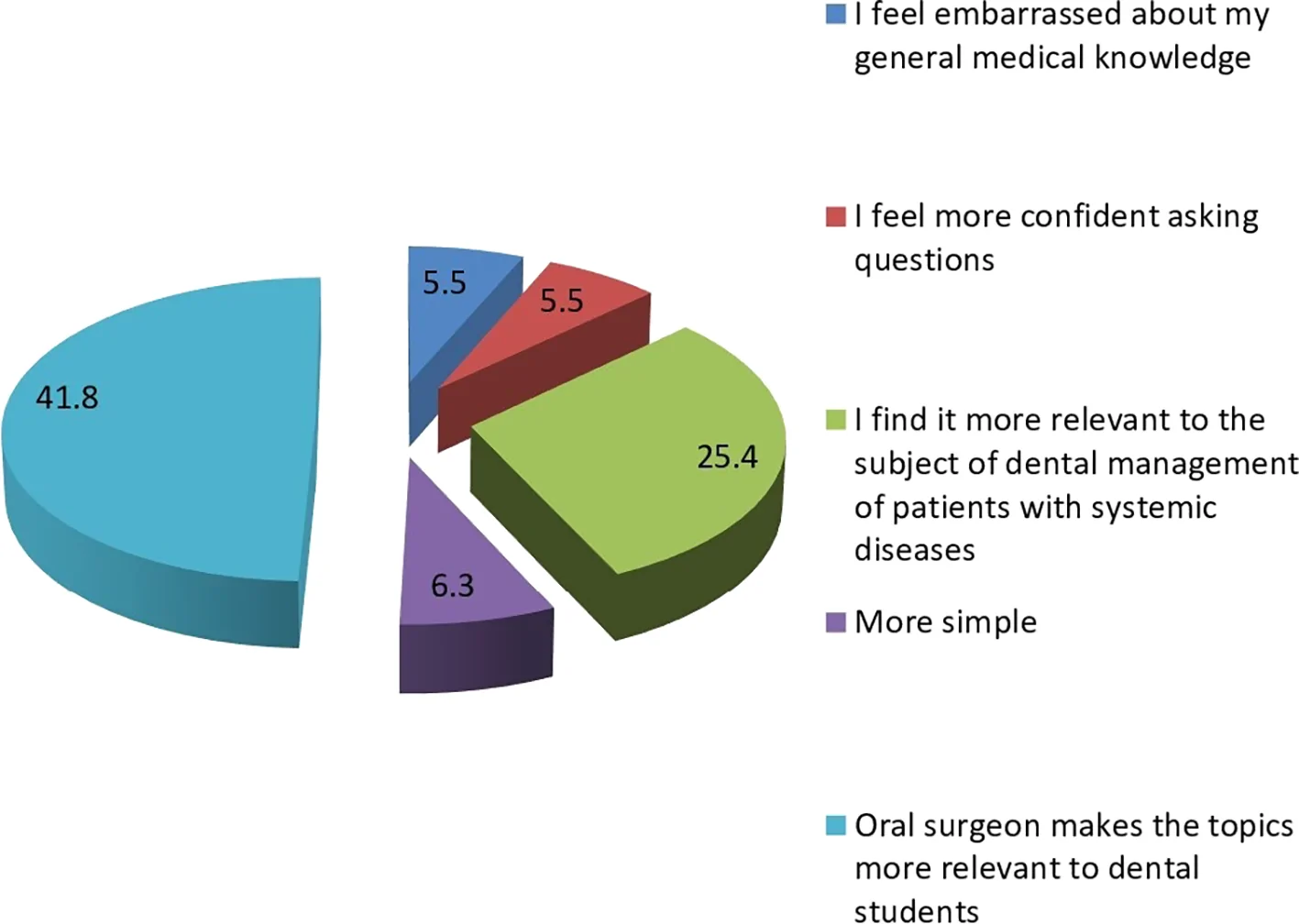
Reasons for preference oral surgeon as a lecturer for general surgery subject.

Figure 3 shows that students who think GS helps better understand OS have a comfortable majority among the participants. However, students think that GS could help to some extent in understanding OS is higher than students who think GS could provide considerable help in this matter. Understandably, the Chi-Square Test showed a highly significant relationship ( *P *= 0.000) between the relevance of GS to OS and the extent to which GS help in understanding OS. Surprisingly, a considerable majority of the students (almost 70%) wished to become oral and maxillofacial OMF surgeons ( Table 1). This wish does not seem to be influenced by the students’ gender. The chi-square Test showed no statistically significant difference (P = 0.313) between males and females in their willingness to become OMF surgeons. General surgery lectures were given by an oral surgeon to approximately two-thirds of the participants. This percentage is comparable to the percentage of students who wish to receive GS lectures by oral surgeons. Similarly, the percentage of students who were given the subject by a physician and the percentage of students who were given general surgery lectures by a physician. The reason for the students’ preferences toward the oral surgeon is illustrated in Figure 4. Around half of the respondents prefer GS to be given by an oral surgeon as he/she makes the subject more relevant to dentistry. Another important reason is related to the fact that GS, when performed by an oral surgeon, helps to better understand the management of systemic diseases from a dental perspective. Other reasons, such as simplicity, embarrassment, and being more comfortable asking questions, share a minority of the remaining responses.

The chi-square Test showed a significant relationship (P = 0.021) between willingness to become an OMF surgeon and the wish to be given a GS subject by an oral surgeon. However, there was no significant relationship between the intention to become an OMF surgeon and the academic staff who gave GS lectures (P = 0.123).

## Discussion

In Iraqi dental schools, both general surgery GS and general medicine are included in the 4th academic 4th year curriculum. During this year, dental students begin their undergraduate clinical training in different dental specialties. Clinical training represents a stressful environment. ^9– 10^ This might cast a shadow over what might be considered as non-dental curricular subjects during academic years, where clinical requirements have an overwhelming burden on both 4th and 5th year dental students. ^11^ In the 4th academic year, a general surgery subject was given to dental students along with oral surgery subjects. It aims to equip dental students with essential knowledge of different surgical topics of shared interest. In this course, students were given lectures on the basic management of surgical patients. General surgical knowledge has been a subject of prime interest in dental education for decades. ^12, 13^ This study aimed to investigate current dental students’ perceptions of general surgery subjects. It seems that general surgery enjoys a declining level of popularity, as reported in this study. This could be related to the students’ perception that general surgery is of value to OMFS. This is supported by the fact that the majority of the participants chose to become OMF surgeons. Dental students’ tendency to choose OMFS as their career has been investigated in several studies. ^14– 17^ According to our study, students’ positive views toward GS subjects do not seem to be influenced by the academic staff who give the lecture, as is evident from this study. Although many students think that the oral surgeon makes the GS topics more relevant to their specialty, some think that the oral surgeon addresses systemic disease and their management from a dental perspective. The notion that surgeons who provide students with the necessary surgical knowledge should have dental expertise is as old as in the 19th century literature. ^18^ It appears that there is a distinction between GS and OMFS in students’ mindsets. Generally, surgical subjects do not address oral and maxillofacial surgical problems. The curriculum of GS as given to Iraqi dental students mainly deals with shock, homeostasis, electrolyte and body fluid balance, management of burns, and general management of surgical wounds. ^8^ This might explain the neutral response of participants to the relationship between GS and oral surgery. Clinical oral surgery training in undergraduate schools is mainly performed in an outpatient setting. Undergraduate students are keener to improve their technical skills compared to their theoretical skills. ^19^ The general surgical subject is provided mainly as theoretical material. Another reason might be related to the fact that theoretical principles in oral surgical practice are mainly directed towards the oral cavity environment. The nature of surgery in the tooth-bearing area has special anatomical, histological, and physiological features.

## Conclusion

General surgery enjoys a reasonable level of popularity among undergraduate senior dental students. This popularity seems to have influenced undergraduate students’ willingness to become oral and maxillofacial surgeons. This, in turn, made students prefer general surgery subjects given by an oral surgeon.

## Ethical approval

The Institutional Review Board (IRB) - Ethical Committee at Ibn Sina University of Medical and Pharmaceutical Sciences declares that your manuscript entitled: “Different Dental Students’ Attitude toward General Surgery Subject?” has been reviewed and ethically approved on (29/10/2024), under the number: ISU.16.1.24.

## Consent statement

Written informed consent obtained from participants.

At the beginning of the questionnaire, this statement was included:

“Dear student, by answering the following questions you agree to participate in this questionnaire based study on dental students’ attitude toward general surgery subject. This study aims to investigate various aspects of general surgery subject education. Your answers will provide very useful insight towards the study project and helps to improve general surgery education in the future.”

## Data Availability

FigShare: Different Dental Students’ Attitude toward General Surgery Subject. https://doi.org/10.6084/m9.figshare.28645892. ^20^ Data are available under the terms of the Creative Commons Attribution 4.0 International license (CC-BY 4.0).
