# The Influence of General Surgery Education on Dental Students: A Cross-Sectional Study in Iraqi Dental Schools

**DOI:** 10.12688/f1000research.163454.2

**Published:** 2026-07-01

**Authors:** Ali Jabbar Alsheakh, Najih Alaridhy, Saif Anmar Badran, Faaiz Alhamdani

**Affiliations:** 1 Dentistry Department, Al Yarmouk University College, Diyala, Iraq; 2 Dental Technologies, Alshaab University, Baghdad, Iraq; 3 Ibn Sina University for Medical and Pharmaceutical Sciences, Baghdad, Baghdad Governorate, Iraq

**Keywords:** Dental health, biomedical knowledge, General surgery

## Abstract

**Background:**

Different dental schools might adopt different educational approaches in relation to general surgery subject. In Iraqi dental schools’ general surgery subject is usually given for the fourth academic year students. To the best of the authors’ knowledge, no previous study tried to investigate undergraduate dental students’ attitude toward different aspects of this subject.

**Methods:**

Google-form questionnaire of three main domains was circulated to fourth- and fifth-year dental students in 3 dental schools. These students are given lectures on general surgery by either a physician or oral surgeon.

**Results:**

Two hundred and sixty-five students participated in this study. most responses were positive toward general surgery subject. Chi-Square Test showed a highly significant relationship ( *P*=0.000) between the relevance of GS to OS and the extent that general surgery helps in understanding oral surgery. The same test showed a significant relationship ( *P*=0.021) between willingness to become OMF surgeon and the wish to be given GS subject by oral surgeon.

**Conclusion:**

General surgery enjoys a reasonable level of popularity among undergraduate senior dental students. This popularity seems to influence the willingness of undergraduate students to become oral and maxillofacial surgeons. This in turn made students prefer general surgery to be given by oral surgeon.

## Introduction

Dental health is inseparable from general medical health. Dental schools incorporate dental, relevant biomedical knowledge to prepare proficient dentists with efficient medical knowledge. ^1^ This aims to integrate the three pillars of dental educations, which are: technical skills, clinical knowledge; motivate, empathize with patient; critical, clinical reasoning, decision making. ^2, 3^ Accordingly, dental curriculum includes almost the same basic biological, and medical knowledge adopted in medical schools with more focus on the head and neck region, and dental apparatus. Dentistry has its uniqueness among other medical profession. ^4, 5^

Of the given subjects for undergraduate dental students are general medicine, and general surgery. General medicine is related to a variety of systemic diseases, which have direct or indirect influence on patients undergoing dental treatment. General surgery, on the other hand, tries to provide dental students with the important surgical knowledge, which is relevant to their medical background and their oral surgical practice. ^6, 7^

Different dental schools might adopt different educational approaches in relation to general surgery subject. In Iraqi dental schools general surgery subject is usually given for the fourth academic year students. ^8^ To the best of the authors’ knowledge, no previous study tried to investigate undergraduate dental students’ attitude to different aspects of this subject.

### Aim of the study

To evaluate the influence of general surgery education on students’ preference for lecturer specialty (oral surgeon vs physician).

## Materials and methods

This cross-sectional study was approved by the Ethical Committee in Ibn Sina University of Medical and Pharmaceutical Sciences (ISU.16.1.24). It is a questionnaire-based study using online Google-form. This questionnaire was circulated to fourth- and fifth-year dental students in 3 dental schools. Sample selected to ensure diverse response from both 4 ^th^ and 5 ^th^ year students in three Iraqi dental schools where general surgery lectures are delivered by oral and maxillofacial surgeon as well as general surgeon. A total of 265 students participated, providing a sufficiently large and representative sample for the study.

All participants were informed about the purpose of the study; their personal information will be kept anonymized; and they have the right to withdraw from the study. This statement was provided at the beginning of the questionnaire. They were informed that answering the questionnaire after reading this statement represents their agreement for participation.

The questionnaire was developed by the first author. To ensure face validity, the questionnaire was reviewed by both oral and maxillofacial surgeon and general surgeon who are actively involved in teaching GS for dental students. Chronbach’s Alfa value (for internal reliability) was 0.615. This is considered acceptable for attitudinal measures, particularly when the number of measured items is low. ^9^ The choice of participants was based on the academic staff that gives the GS for the fourth year. Fourth- and fifth-year students were included, as fifth year students might have additional input. Fifth year students are given theoretical lectures mainly on maxillofacial surgery items, which expand their knowledge and appreciation toward GS subject.

The questionnaire covered different aspects of general surgery GS subject in relation to oral surgery OS knowledge and practice. The questionnaire items are divided into 3 domains; demographic domain, which includes general information about the participants. The second domain is related to GS subject, whereas the third domain focuses on the relation between GS and OS subjects. The questionnaire items are shown in Appendix 1.

Both descriptive and inferential statistics were used. Chi-Square Test was used to determine the statistical relationship between nominal variables. SPSS Ver.23 was used for statistical analysis with a *P* value <0.05 was considered for statistical significance.

## Results

Two-third of the respondents were females. Males constitute the remaining one-third. Students from three Iraqi dental schools participated in this study. Two dental schools are private dental schools (Al-Yarmouk University college, and Al-Hikmah University College), and one governmental dental school (Ibn Sina University of Medical and Pharmaceutical Sciences. All the three dental schools based in Baghdad, Iraq. Table 1 provides the demographics of the study.

**Table 1.** Study demographic data.

students’ gender	No.	%
male	84	32.8
female	172	67.2
students’ academic year	No.	%
fourth year	160	62.5
fifth year	96	37.5
dental school	No.	%
Ibn Sina University	95	37.1
Al-Yarmouk UC	76	29.7
Al-Hikmah UC	85	33.2
academic staff specialty	No.	%
oral surgeon	164	64.1
physician	92	35.9
Who should give GS lecture	No.	%
Oral surgeon	175	68.4
physician	81	31.6
The wish to be OMFS	No.	%
YES	177	69.1
NO	79	30.9

**Figure 1.**
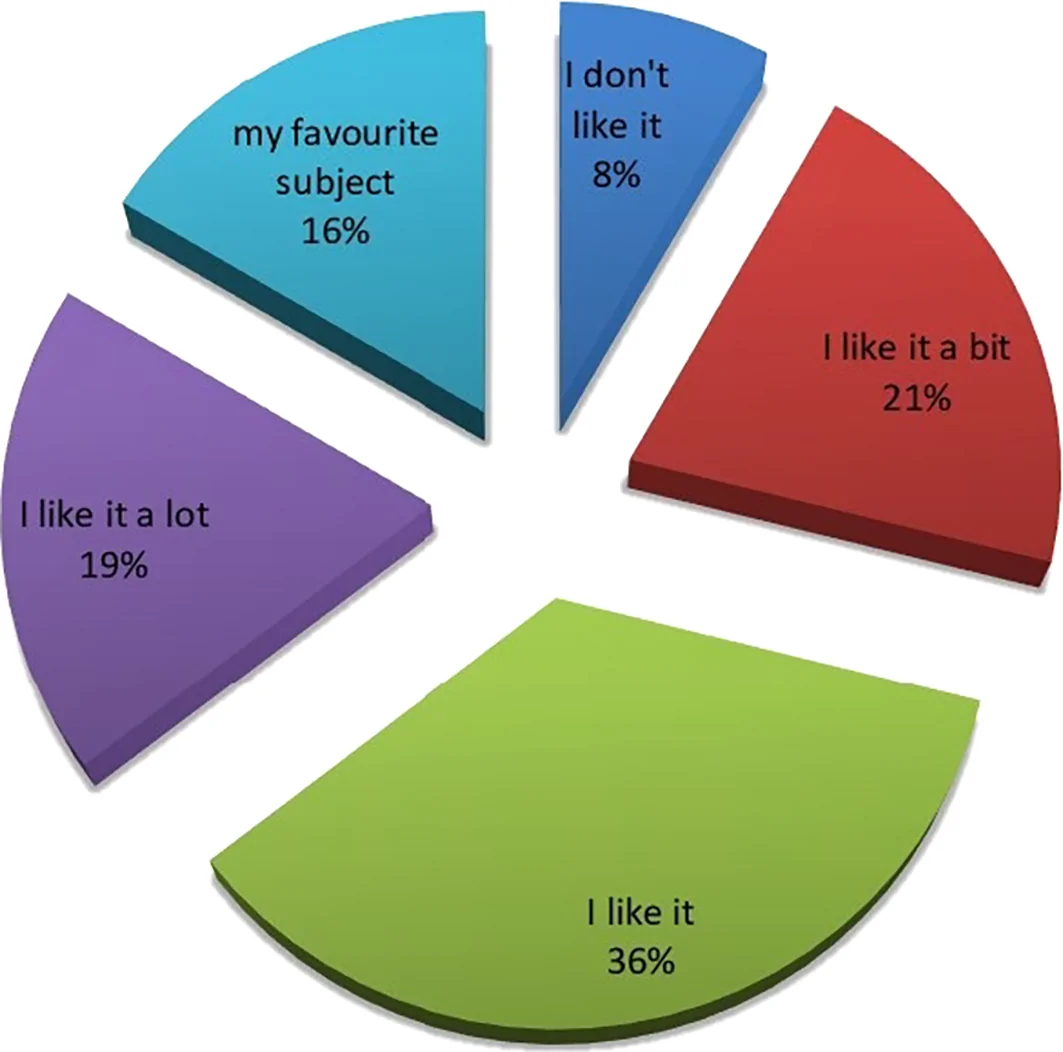
The level of likeness of general surgery.

As shown in Figure 1, students’ preference toward general surgery GS subject varies. However, the majority of responses were positive. There are, even, students who consider the GS subject as their favorite. Only a small minority of students do not seem to like the subject. The comparison between the percentage of response “my favorite subject” and “I don’t like it” might give a hint on the likeliness of the subject.

**Figure 2.**
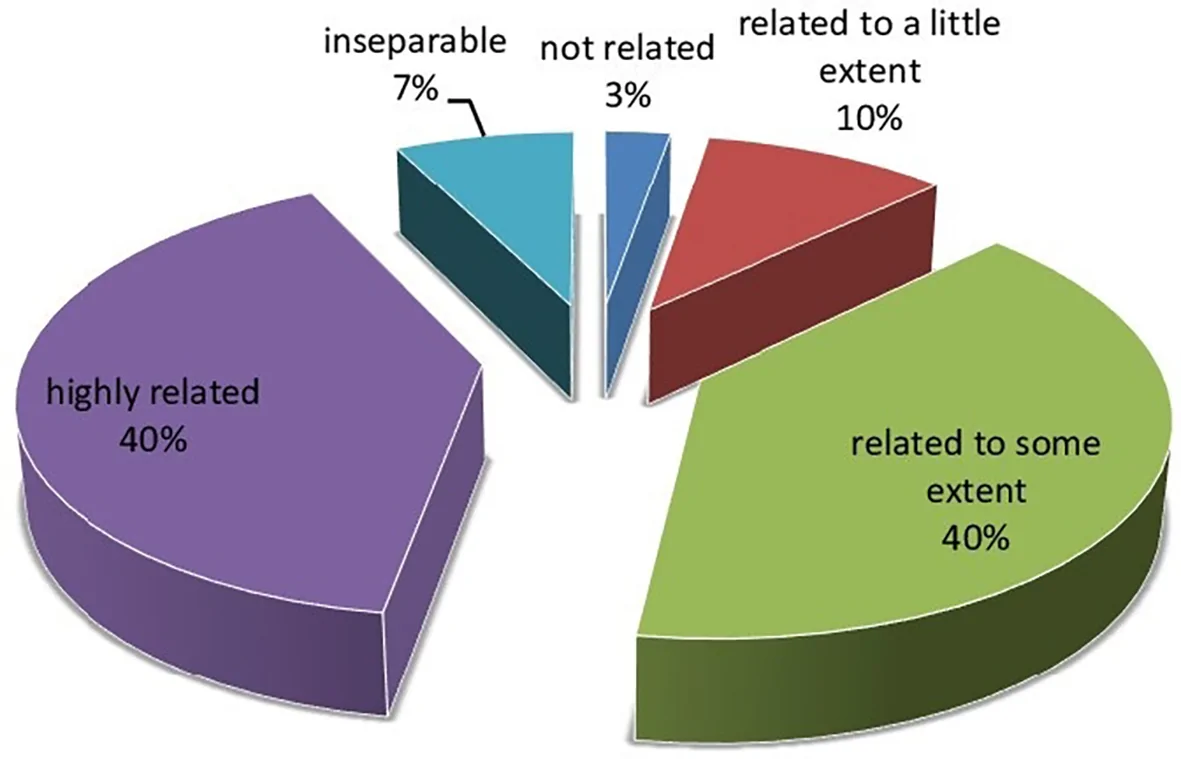
General surgery subject relevance to oral surgery subject.

An overwhelming majority of the students think GS is related to oral surgery, to a variable degree though. Figure 2 also shows that almost half of the students believe in the high relationship between the subjects. Very small minority of students find GS irrelevant to OS.

**Figure 3.**
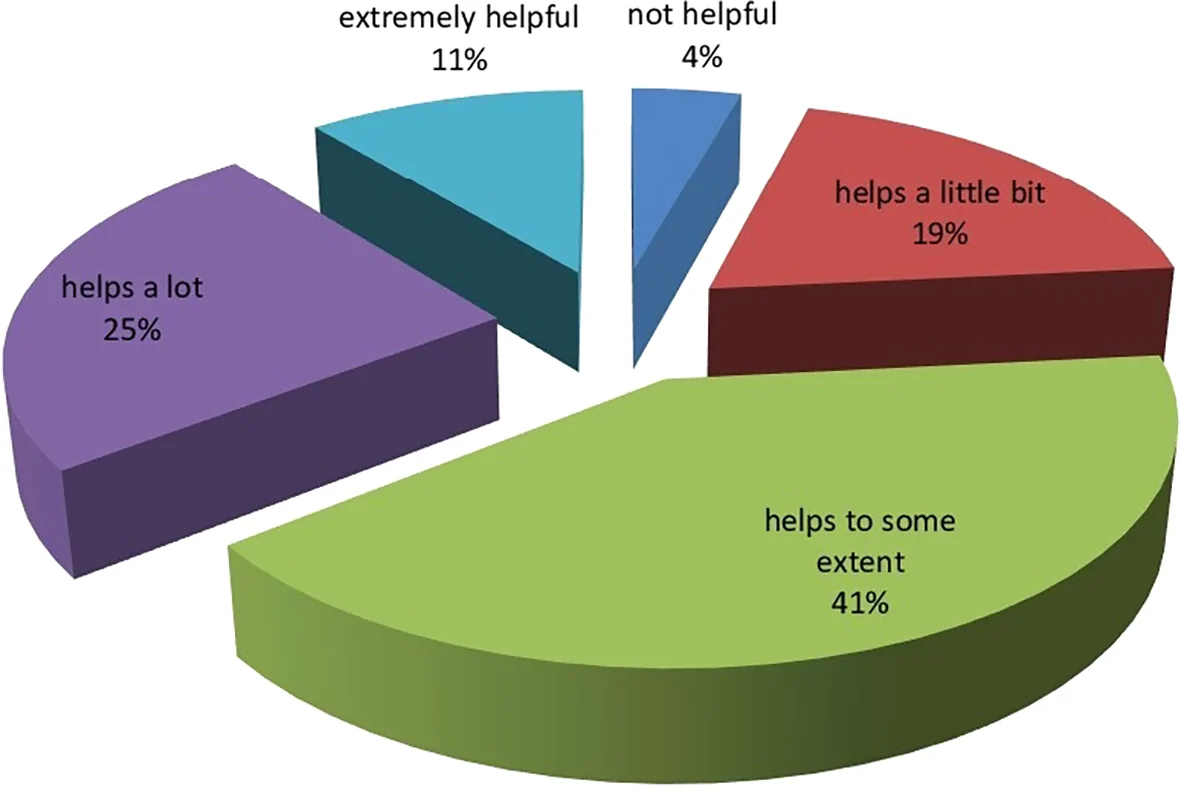
The perceived help provided by general surgery subject in understanding oral surgery.

Figure 3 shows that students think GS helps for better understanding of OS has the comfortable majority among the participants. However, students think that GS could help to some extent in understanding OS is higher than students who think GS could provide considerable help in this matter. Understandably, Chi-Square Test showed a highly significant relationship ( *P* = 0.000) between the relevance of GS to OS and the extent that GS help in understanding OS.

Surprisingly, a considerable majority of students (almost 70%) wish to become oral and maxillofacial OMF surgeons ( Table 1). This wish does not seem to be influenced by students’ gender. Chi-Square Test showed no statistically significant difference ( *P* = 0.313) between males and females in their willingness to become OMF surgeons.

General surgery lectures are given by oral surgeons to around two-thirds of the participants. This percentage is comparable to the percentage of students who wish to be given GS lecture by oral surgeon. Similarly, the percentages of students who are given the subject by a physician and the percentage of the students who are given general surgery lectures by a physician.

**Figure 4.**
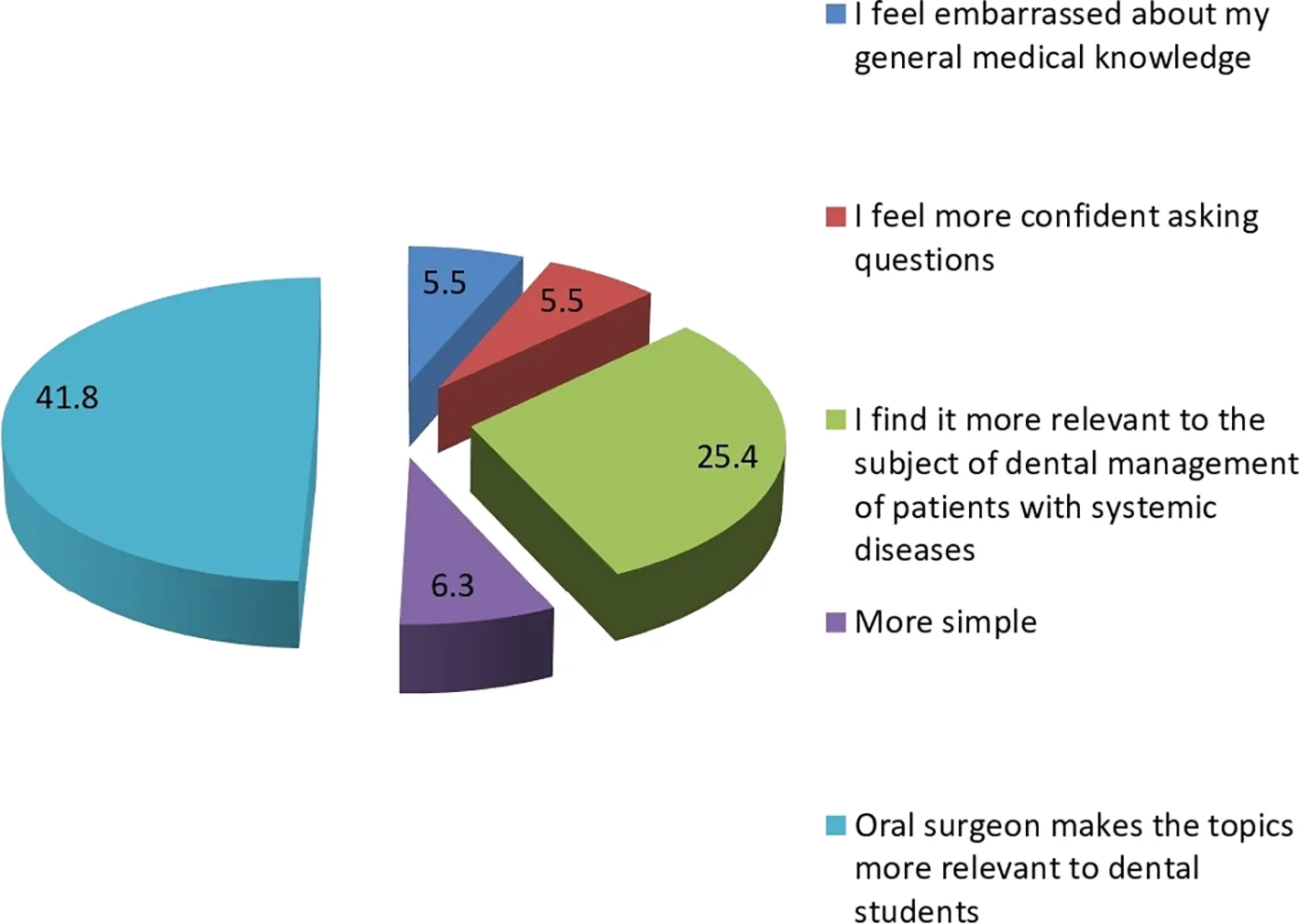
Reasons for preference oral surgeon as a lecturer for general surgery subject.

The reason students’ preference toward the oral surgeon is illustrated in Figure 4. Around half of the respondents prefer GS subject to be given by oral surgeons as he/she makes the subject more relevant to dentistry. another important reason is related to the fact that GS when given by oral surgeon helps more in understanding the management of systemic diseases from dental perspective. Other reasons. such as simplicity; embarrassment; and being more comfortable asking questions share the minority of the remaining responses.

Chi-Square Test showed a significant relationship ( *P* = 0.021) between willingness to become OMF surgeon and the wish to be given GS subject by oral surgeon. However, there was no significant relationship between the intention to become OMF surgeon and the academic staff who give the GS lectures ( *P* = 0.123).

## Discussion

In Iraqi dental schools, both general surgery GS and general medicine are included in the 4 ^th^ academic 4 ^th^ year curriculum. During this year dental students start their undergraduate clinical training in different dental specialties. This clinical training represents a stressful environment. ^10, 11^ This might cast a shadow over what might be considered as non-dental curricular subjects during academic year where clinical requirement has its overwhelming burden on both 4 ^th^ and 5 ^th^ year dental students. ^12^

In the 4 ^th^ academic year, general surgery subject is given to dental students alongside oral surgery subject. It aims to equip dental students with essential knowledge on different surgical topics of shared interest. In this subject, students are given lectures on basic management of surgical patients. General surgical knowledge has been a subject of a prime interest in dental education for decades. ^13, 14^

This study aims to investigate the current dental students’ perception toward general surgery subject. It seems that general surgery enjoys a descent level of popularity as reported in this study. This could be related to students’ perceptive notion that general surgery is of value to OMFS. This is supported by the fact that most of the participants’ choice to become OMF surgeons.

Dental students’ tendency to choose OMFS as their career has been investigated by several studies. ^15– 18^ According to our study, students’ positive views toward GS subject do not seem to be influenced by the academic staff who give the lecture, as is evident in this study. Although many students think that the oral surgeon makes the GS topics more relevant to their specialty and some think that the oral surgeon addresses systemic disease and their management from dental perspective. The notion that the surgeon who provide students with the necessary surgical knowledge should have dental expertise is as old as the 19 ^th^ centuries literature. ^19^

It appears that there is a distinction between GS and OMFS in the students’ mindset. General surgery does not address oral and maxillofacial surgical problems. This fact has been acknowledged by a study conducted by Nadia Shahid *et al.* In their qualitative study they reported that dental students think that GS material should be tailored to be more relevant for emergency care and maxillofacial surgery. ^20^

Clinical oral surgery training in the undergraduate school is performed mainly under an out-patient setting. Undergraduate students are more keen to improve their technical skills than their theoretical skills. ^21^ General surgery is provided mainly as theoretical material. Theoretical principles in oral surgical practice are mainly directed to oral cavity environment. The nature of the surgery in tooth bearing area enjoys special anatomical, histological, and physiological features.

The curriculum of GS as given to Iraqi dental students mainly deals with shock, homeostasis, electrolytes and body fluid balance, management of burns, and general management surgical wounds. ^8^ This might explain the participants’ neutral response to the relation of GS and oral surgery. In Nadia Shahid qualitative study, participants stated that such topics are necessary. However, in-depth material might not be necessary. ^20^ This needs to be considered within the stressful overloaded environment of undergraduate dental education. This fact has been mentioned in other studies. ^12, 21^

To the best of the authors’ knowledge this is the first quantitative study tries to investigate GS subject for undergraduate dental education. This might reflect the marginalization of this field in dental education. Dental curricula seem to be more focused on oral surgery. ^22, 23^ Undergraduate dental students are mainly trained to master outpatient oral surgical procedures.

There is an important limitation of this study. It was not feasible to conduct a comparative analysis in which both oral surgeons and general surgeons delivered lectures to the same group of students. In Iraqi dental schools, the General Surgery course is taught by either an oral surgeon or a general surgeon. This variation in teaching background may understandably influence students’ attitudes, it also reflects the real-world educational context in which these students are trained. Therefore, the authors believe that students’ perspectives remain valuable, as they provide authentic insights into how different instructional approaches shape learning experiences.

## Conclusion

General surgery enjoys a reasonable level of popularity among undergraduate senior dental students. This popularity seems to influence the willingness of undergraduate students to become oral and maxillofacial surgeons. This in turn made students prefer general surgery to be given by oral surgeons.
