# Supplementary material for: The Influence of General Surgery Education on Dental Students: A Cross-Sectional Study in Iraqi Dental Schools
Source: F1000Res. 2026 Aug 24;14:521. Originally published 2025 May 27. [Version 3] doi: 10.12688/f1000research.163454.3 (PMC13560059; doi:10.12688/f1000research.163454.3)
Supplement: Peer review history

# Reviewer report: Reviewer response for version 3

**DOI:** 10.5256/f1000research.208873.r514044

**Published:** 2026-08-25

**Authors:** Abdalbseet A Fatalla

**Affiliations:** 1 University of Baghdad, Baghdad, Baghdad Governorate, Iraq

Dear Editor

 All necessary corrections done

 Regards

Is the work clearly and accurately presented and does it cite the current literature?

Yes

If applicable, is the statistical analysis and its interpretation appropriate?

I cannot comment. A qualified statistician is required.

Are all the source data underlying the results available to ensure full reproducibility?

Yes

Is the study design appropriate and is the work technically sound?

Yes

Are the conclusions drawn adequately supported by the results?

Yes

Are sufficient details of methods and analysis provided to allow replication by others?

Yes

Reviewer Expertise:

dentistry, biomaterials, dental implants,

I confirm that I have read this submission and believe that I have an appropriate level of expertise to confirm that it is of an acceptable scientific standard.

# Reviewer report: Reviewer response for version 2

**DOI:** 10.5256/f1000research.198916.r507442

**Published:** 2026-08-08

**Authors:** Abdalbseet A Fatalla

**Affiliations:** 1 University of Baghdad, Baghdad, Baghdad Governorate, Iraq

The abstract addresses a relevant and under-investigated educational topic: undergraduate dental students’ perceptions of general surgery (GS) and its relationship to oral and maxillofacial surgery (OMFS). The multicenter approach involving three dental schools is a strength. 

  however some comments should be addressed by the authors:

 1. Explain why the way GS is taught (physician vs oral surgeon) might influence students’ perception of the subject and their career interest.

 2. questionnaire should be sufficiently described

 3. Conclusion need modification

Is the work clearly and accurately presented and does it cite the current literature?

Yes

If applicable, is the statistical analysis and its interpretation appropriate?

I cannot comment. A qualified statistician is required.

Are all the source data underlying the results available to ensure full reproducibility?

Yes

Is the study design appropriate and is the work technically sound?

Yes

Are the conclusions drawn adequately supported by the results?

Yes

Are sufficient details of methods and analysis provided to allow replication by others?

Yes

Reviewer Expertise:

dentistry, biomaterials, dental implants,

I confirm that I have read this submission and believe that I have an appropriate level of expertise to confirm that it is of an acceptable scientific standard.

# Reviewer report: Reviewer response for version 2

**DOI:** 10.5256/f1000research.198916.r497881

**Published:** 2026-07-06

**Authors:** Atheer Talib Jiboon

**Affiliations:** 1 Mustansiriyah University, Baghdad, Baghdad Governorate, Iraq

After the corrections; the research is well organized.

 The title, validity of the questions, and discussion section are clarified.

 I have no further comments

Is the work clearly and accurately presented and does it cite the current literature?

Partly

If applicable, is the statistical analysis and its interpretation appropriate?

I cannot comment. A qualified statistician is required.

Are all the source data underlying the results available to ensure full reproducibility?

Yes

Is the study design appropriate and is the work technically sound?

Yes

Are the conclusions drawn adequately supported by the results?

Yes

Are sufficient details of methods and analysis provided to allow replication by others?

Yes

Reviewer Expertise:

Oral and Maxillofacial surgery

I confirm that I have read this submission and believe that I have an appropriate level of expertise to confirm that it is of an acceptable scientific standard.

# Reviewer report: Reviewer response for version 1

**DOI:** 10.5256/f1000research.179808.r461577

**Published:** 2026-03-23

**Authors:** Atheer Talib Jiboon

**Affiliations:** 1 Mustansiriyah University, Baghdad, Baghdad Governorate, Iraq

The topic of this study is interesting and relevant, as it explores the integration between general surgery and oral surgery within the dental curriculum. This is an important area, particularly in improving interdisciplinary education for dental students.

 However, several points need to be addressed:

**1. Title**

 The current title requires refinement for clarity and specificity. It is recommended to revise it to:

**“The Influence of General Surgery Education on Dental Students: A Cross-Sectional Study in Iraqi Dental Schools.”**

**2. Aim of the Study**

 The study aim should be more concise and clearly structured. It can be reformulated as:

 “To evaluate the influence of general surgery education on oral surgery training and on dental students’ future specialty preferences.”

**3. Methods Section**

 The methods section is incomplete and requires additional details. Specifically:

- The sample size must be clearly stated.
- The statistical analysis methods used should be described in detail.
- To ensure the validity of the questions; if there was pilot study; it should be mentioned.

If other academics (experts) check the questions, it should be mentioned and that will also increase the validity.

**4. Results Section**

 In Table 1, the findings regarding who should deliver general surgery lectures show that approximately one-third of students prefer general surgeons. Interestingly, this corresponds to the proportion of students who were actually taught by general surgeons.

 All participants should have been exposed to teaching by both general surgeons and oral surgeons. Without such standardization, it is recommended that this question—and its associated results—be omitted, as it may introduce bias and limit the validity of the conclusions.

 The above controversy is also implied on figure 4

**5. Discussion Section**

 The discussion requires further development. Specifically:

- Greater elaboration on the study findings with relation to the published literatures is needed.
- Focus more directly on the study aims, particularly:
  - The influence of general surgery education on oral surgery training, try to reference other studies that support this result.
  - Students’ preferences regarding future specialization

Strengthening this section will enhance the scientific value and contextual relevance of the study.

**Overall**, the study addresses an important educational topic; however, revisions are necessary to improve methodological clarity, and depth of discussion before it is suitable for indexing/

Is the work clearly and accurately presented and does it cite the current literature?

Partly

If applicable, is the statistical analysis and its interpretation appropriate?

I cannot comment. A qualified statistician is required.

Are all the source data underlying the results available to ensure full reproducibility?

Yes

Is the study design appropriate and is the work technically sound?

Yes

Are the conclusions drawn adequately supported by the results?

Yes

Are sufficient details of methods and analysis provided to allow replication by others?

Yes

Reviewer Expertise:

Oral and Maxillofacial surgery

I confirm that I have read this submission and believe that I have an appropriate level of expertise to confirm that it is of an acceptable scientific standard, however I have significant reservations, as outlined above.

# Reviewer report: Reviewer response for version 1

**DOI:** 10.5256/f1000research.179808.r394469

**Published:** 2025-07-03

**Authors:** Sayem Anwarhussain Mulla

**Affiliations:** 1 Dentistry, Independent Researcher, Kalyan, Maharashtra, India

The topic is novel. However, it lacks proper structuring. The authors are confused to focus on what. 2-3 different aims are given. And the given aims are not properly formatted. The validation of questionnaire is not mentioned properly (face validation/subject expert validation). Statistics are also not in-depth, although for the aim they are fine but the authors can do more in-depth analysis (depends on editor's decision). Discussion is too concise, kindly expand more. Recent references are lacking, try if you could get it. Overall, the topic is good and the article holds promise but these changes are necessary for a bigger and better impact.

Is the work clearly and accurately presented and does it cite the current literature?

Partly

If applicable, is the statistical analysis and its interpretation appropriate?

I cannot comment. A qualified statistician is required.

Are all the source data underlying the results available to ensure full reproducibility?

Yes

Is the study design appropriate and is the work technically sound?

Partly

Are the conclusions drawn adequately supported by the results?

Partly

Are sufficient details of methods and analysis provided to allow replication by others?

Yes

Reviewer Expertise:

Dentistry

I confirm that I have read this submission and believe that I have an appropriate level of expertise to confirm that it is of an acceptable scientific standard, however I have significant reservations, as outlined above.
